# Modeling time to death for under-five children in Malawi using 2015/16 Demographic and Health Survey: a survival analysis

**DOI:** 10.1186/s41043-024-00538-y

**Published:** 2024-04-03

**Authors:** Assa Mulagha-Maganga, Lawrence Kazembe, Martin Ndiragu

**Affiliations:** 1 African Center of Excellence in Agriculture Policy Analysis, Lilongwe University of Agriculture and Natural Resources, Lilongwe, Malawi; 2 Regional Office for Africa, World Health Organization (WHO), Cite du Djoue, P.O Box 06, Brazzaville, Congo; 3 Department of Mathematical Sciences (Biostatistics), University of Malawi, Zomba, Malawi; 4 Office of Evaluations, Everest Intelligence Consult Ltd, Meanwood Kamwena, Chamba Valley, Lusaka, Zambia

**Keywords:** Under-five child mortality, Socioeconomic factors, Time to death, Malawi

## Abstract

**Background:**

Malawi has one of the highest under-five mortality rates in Sub Sahara Africa. Understanding the factors that contribute to child mortality in Malawi is crucial for the development and implementation of effective interventions to reduce child mortality. The aim of this study is to use survival analysis in modeling time to death for under-five children in Malawi. In turn, identify potential risk factors for child mortality and inform the development of interventions to reduce child mortality in the country.

**Method:**

This study used data from all births that occurred in the five years leading up to the 2015/16 Malawi Demographic and Health Survey. The Frailty hazard model was applied to predict infant survival in Malawi. In this analysis, the outcome of interest was death and it had two possible outcomes: "dead" or "alive". Age at death was regarded as the survival time variable. Infants who were still alive at the time of the study as of the day of the interview were considered as censored observations in the analysis.

**Results:**

A total of 17,286 live births born during the 5 years preceding the survey were analysed. The study found that the risk of death was higher among children born to mothers aged 30–39 and 40 or older compared to teen mothers. Infants whose mothers attended fewer than four antenatal care visits were also found to be at a higher risk of death. On the other hand, the study found that using mosquito nets and early breastfeeding were associated with a lower risk of death, as were being male and coming from a wealthier household.

**Conclusion:**

The study reveals a notable decline in infant mortality rates as under-five children age, underscoring the challenge of ensuring newborn survival. Factors such as maternal age, birth order, socioeconomic status, mosquito net usage, early breastfeeding initiation, geographic location, and child's sex are key predictors of under-five mortality. To address this, public health strategies should prioritize interventions targeting these predictors to reduce under-five mortality rates.

## Introduction

The UN General Assembly on Sustainable Development Goal (SDG) 2015 called for all countries to at least reach under-5 mortality rate (U5MR) of 25 deaths per 1000 livebirths and 12 deaths per 1000 livebirths for neonatal mortality rate (NMR) by 2030 [22]. The under-5 mortality rate is defined as the probability of a child dying between birth and exactly 5 years of age, expressed per 1,000 live births [23]. In the last 3 decades, the world has made notable progress in ensuring a child’s survival in its first 5 years. Compared to the 1990s, children born in 2020 have better survival chances of reaching 5 years. Between 1990 and 2020, the under-5 mortality rates have significantly reduced by 59%, from a rate of 93 deaths per 1000 live deaths. This interprets to 1 in 11 in 1990 to 1 in 27 in 2020. UNICEF estimates 13,800 under-5 daily deaths in 2020 [17, 25].

**Fig. 1:**
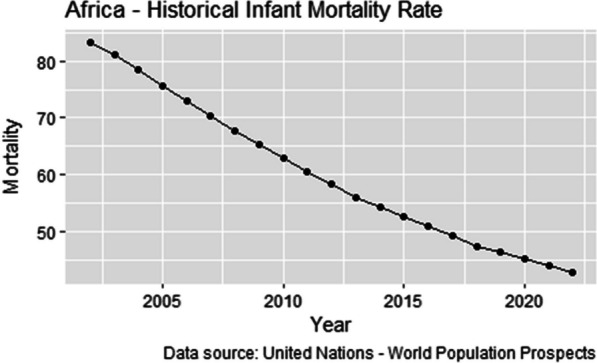
Trends in infant mortality in Africa

Infant and child mortality rates are basic indicators of a country’s socioeconomic situation and quality of life [18]. Despite the global under-5 mortality rates significantly decreasing to 37 deaths per 1000 live births in the last few decades, sub-Saharan Africa continues to have the highest under-5 mortality rates in the world [17]. Currently, the infant mortality rate in Africa is 42.7 deaths per 1000 live births, a 2.67% decline from 2021. The trend has been constantly declining for the past two decades and the mortality rates have almost halved over the same period Fig. 1. This is not surprising as the reducing infant mortality has been one key goal of global priority.

There has been increasing investments to reduce the infant mortality. Huge investments have been made in development and increasing access to vaccines, promotion of infant and young feeding practices, improved pediatric and heath care [21]. While overall, the statistics show a persistent decline in infant mortality rates, infants’ deaths are driven by economic hardships both in-country and across. This is one of the reasons for high infant deaths in developing countries compared to developed countries [21]. Malawi, one of the poorest countries in Africa, has statistic for under-five mortality rate in general that exceeds the average for Africa. Over the period 2010 to 2015, the neonatal mortality rate was 27 deaths per 1,000 live births. This means that 1 in every 37 children in Malawi dies in the first month of life. The infant mortality rate was higher, with 42 deaths per 1,000 live births; this means that 1 in every 24 children dies before celebrating their first birthday. The under-5 mortality rate of 63 deaths per 1,000 live births translates to 1 of every 16 children dying before their fifth birthday. Under-5 mortality declined from 234 deaths per 1,000 live births in 1992 to 63 deaths per 1,000 live births in 2015 representing a 73% decrease.

Recent studies conducted in various spaces of Africa have identified socio-demographic, maternal and child characteristics as key drivers of under-five child survival [19]. The socio-economic differences result in different levels of constraints to access quality health services and prioritization of the child’s health. A poor mother is more likely not to seek quality health services for her child as compared to a well-off mother. Poor parents are more unlikely to afford quality health care, are more likely to delay seeking treatment, or are more likely to resort to traditional medicines for their infants [8]. Ng’ambi et al. [13] also shows that there is less health seeking behavior among the poor households. Children from multiple-birth mothers compared to children from single-birth mothers are up to 5 times more likely to die within 59 months of their birth [5]. Geographical location and region may also play a role on the likelihood of under-five mortality. In a country, some areas may have more well-developed and equipped health services than other areas. This will in turn affect the ability of the mother to access postnatal and prenatal services. The distance and available services will also have an effect on the quality of health the mother and child will access after birth. Available evidence suggests that mothers who deliver in health facilities have lower chances of reporting child death compared to those who deliver at home [7].

There are limited studies conducted to specifically investigate the determinants of under-five mortality in Malawi. Ntenda et al. [15] identified factors such as socioeconomic, maternal, cultural, household, environmental, biological, and health service utilization as determinants of under-five mortality in Malawi. Malnutrition, pneumonia, birth asphyxia, diarrhea and malaria, immunization, breastfeeding, maternal age, maternal education level, and sanitation have also been identified as the main cause of infant deaths. The study applied a logistic regression to study the association between these covariates and the mortality outcomes but did not explore the survival dynamics as can be leveraged from survival analysis techniques. A logistic model on the other hand assumes that probability of death events is the same on the continuum of year zero to year 5 for the under-five. Nevertheless, the factors explored remain important. Given that low-income countries contribute more to under-five mortality, our study remains very relevant both at country scale and globally. As global efforts are pulled together to achieve Sustainable Development Goal (SDG) of reducing preventable deaths among under-five children, country specific evidence for the right mix of actions to reduce child mortality is required. Our study, therefore seeks to estimate time to death in early years of life for under-five children and the associated risk factors by applying the survival analysis methods using a nationally representative Demographic Health Survey data for Malawi. This will aid understanding of when and why infant deaths are likely to occur and what strategies can be key in reducing the same.

## Methodology

### Data sources

The study uses secondary data from the 2015/16 Malawi’s Demographic Health Survey (MDHS) as part of national surveys implemented by the National Statistical Office for Malawi over a 4-month period, from 19 October 2015 through 17 February 2016. The sampling frame used for the 2015/16 MDHS was the frame of the Malawi Population and Housing Census (MPHC), conducted in Malawi in 2008. The 2015/16 MDHS sample was stratified and selected in two stages. Each district was stratified into urban and rural areas. In the first stage, 850 standard enumeration areas (SEAs), including 173 SEAs in urban areas and 677 in rural areas, were selected with probability proportional to the SEA size and with independent selection in each sampling stratum. In the second stage of selection, a fixed number of 30 households per urban cluster and 33 per rural cluster were selected with an equal probability systematic selection from the newly created household listing. A total of approximately 24,562 women were interviewed. The Woman’s Questionnaire collected information from eligible women age 15–49 who were asked different sets of questions. Of interest for this study was background characteristics; Reproduction: children ever born, birth history; Maternal and child health, breastfeeding, and nutrition: prenatal care, delivery, postnatal care, breastfeeding and complementary feeding practices, vaccination coverage.

A total of 17,286 live birth were recorded over a five-year recall period preceding the survey and these were the candidate cases for infant mortality analyses. The women questionnaire included questions on whether the women had ever born a child and the current age of the child. This information was used to subset the data of children born within the last 5 years prior to the survey. The children included for analysis were those born between 2010 to 2015 for the women interviewed in 2015, and between 2011 to 2016 for those women interviewed in 2016.

Variables: The outcome variable in this study was time to death of an under-five child. Death happening between anytime from birth to 59 months was considered as an event. Children surviving 59 months were censored. Death was not characterized, regardless of any cause, any occurrence of death for the under-five child was considered an event. A number of covariates were introduced to control for drivers of deaths. Building on the previous studies as earlier reviewed this study included the following covariates: Mothers age, Mother’s education level, Wealth Index, Sleeping in treated net, Breastfeeding, Place of birth, Birth weight of the child, antenatal visits.

### Statistical estimation procedure

To provide contextual understanding of the variables included in the analysis, a univariate analysis was conducted on the socioeconomic, demographic factors and child survival. Chi-square test of independence was used to test bivariate relationship between covariates and survival/failure outcomes. Child survival was estimated using:

1 $${s}_{t}=\prod_{ti\le t}1-\frac{{d}_{t}}{{n}_{t}}$$

where *t*_i_ is duration of a child at any point of the 59 months period, *d*_t_ is mortality event up to point *t, n*_t_ is the number of children that are at risk of mortality spell just before *t*_*i.*_ [6].

The cox-proportion hazard model was used for multivariate analysis. The cox models determine the probability of event happening over a given interval which is given as the ratio of survival or hazard probabilities. It reflects the length of time a child survived before dying. The inclusion of covariates necessitates computation of how often death occurs in one group compared to the reference group [12]. The Cox proportional hazards model was fitted as follows:

2 $$\lambda \left(t|x\right)={\lambda }_{0}\left(t\right){\text{exp}}(\beta x)$$

where $\lambda \left(t|x\right)$ is the hazard function for the child living up to less than 59 months. The hazard is a function of some unspecified “baseline hazard ${\lambda }_{0}\left(t\right)$ and a set of covariates defined by *X*, $\beta$ is a coefficient vector for various covariates included in the model. The covariates act to multiply the baseline hazard in a time-independent manner [3]. From this model, we derive the hazard ratios. The time-varying coefficient was fitted by extending above basic model as:

3 $$\lambda \left(t|z(t)\right)={\lambda }_{0}\left(t\right){\text{exp}}(\beta x+\gamma Xg(t))$$

where $\beta$ and $\gamma$ are coefficients of time-fixed and time-varying covariates, respectively [26]. To model heterogeneity, shared frailty model was fitted. A frailty model includes, in the hazard function, the value of an additional unmeasured covariate, the frailty, denoted by $\gamma$, yielding a hazard function as using:

4 $${\lambda }_{ij}(t)={\lambda }_{0}\left(t\right){\text{exp}}(\beta {x}_{ij}+{\delta }_{i})$$

where ${\lambda }_{ij}\left(t\right)$ is the hazard function for the jth individual belonging to *i* th cluster, ${\lambda }_{0}\left(t\right)$ is the baseline hazard at time t, ${x}_{ij}$ is the vector of *k* covariates and ${\delta }_{i}$ is the random effect for the ith cluster [14]. We assume that that the frailty is independent of any censoring that may take place. Because the hazard cannot be negative, distributions must have only positive values. This and other technical issues have led, most frequently, to the use of the Gamma distribution (i.e., a model that assumes that the frailties represent a sample from a Gamma distribution with mean equal to 1 and variance parameter 9). To avoid imposing inappropriate distribution on the frailty, we test it under gamma and inverse-gamma distribution and select the one that is more suited to the data based on smallest Akaike Information Criterion values. Similarly, the baseline hazard can assume various distributions. Hence, in our specification we test the baseline hazard under several distributions including Exponential, Weibull, Loglogistic, Lognormal, Gompertz, Exponential, Weibull, Loglogistic, Lognormal, Gompertz. We also select the model with the smallest Akaike Information Criterion value.

The parameter $\beta$ is found by maximizing the partial likelihood. In order to formulate the partial likelihood, the* f* unique failure times are ordered increasingly ${t}_{0i}$ < ··· < ${t}_{i}$ and *j*(i) is the index of the sample failing at time t_i_. Let ${\mathbf{x}}_{\mathbf{i}}$ be the row vector of covariates for the time interval (${t}_{0i}$*;* ${t}_{i}$] for the *i*th observation in the dataset *i* = 1*, …, N*. We use a method that obtains parameter estimates, $\widehat{\beta }$, by maximizing the partial log-likelihood function for the Cox model:

5 $${\text{log}}\mathcal{L}(\beta )=\sum_{j=1}^{D}\left[\sum_{i\in j}\beta \top {x}_{i}-{d}_{j}{\text{log}}\left\{\sum_{k\in {R}_{j}}{\text{exp}}\left(\beta \top {x}_{k}\right)\right\}\right]$$

where *j* indexes the ordered death times *t*(*j*), *j* = 1*,..., D*; *Dj* is the set of *d*_*j*_ observations that fail at *t*(*j*); *d*_*j*_ is the number of failures at *t*(*j*); and *R*_*j*_ is the set of children *k* that are at risk at time *t*(*j*) (that is, all *k* such that *t*_0*k*_ < *t*(*j*) ≤ *t*_*k*_). This formula for ${\text{log}}\mathcal{L}(\beta )$ is for unweighted data and handles ties by using the Peto–Breslow approximation [2, 16], which is the default method of handling ties. The method treats efficient score residuals as analogs to the log-likelihood scores one would find in fully parametric models. Tied values are handled using Breslow approach as:

6 $${\text{log}}{L}_{breslow}=\sum _{j=1}^{D}\sum _{i\in {D}_{j}}\left[{\widetilde{w}}_{i}\left({x}_{i}\beta +{{\text{offset}}}_{{\text{i}}}\right)-{\widetilde{w}}_{i}{\text{log}}\left\{\sum _{l\in {R}_{j}}{\widetilde{w}}_{i}l {\text{exp}}(xl \beta {+{\text{offset}}}_{{\text{i}}})\right\}\right]$$

where *w*_*i*_ are the weights. In the log likelihood for the Breslow method, ${\widetilde{w}}_{i}={w}_{i}\times N/\sum {w}_{i}$ when the model is fit using probability weights, and ${\widetilde{w}}_{i}={w}_{i}$ when the model is fit using frequency weights or importance weights. Calculations for the exact marginal log likelihood (and associated derivatives) are obtained with 15-point Gauss–Laguerre quadrature. The method provides approximation of the exact marginal log likelihood. While the Efron approximation is a better (closer) approximation, but the Breslow approximation is faster.

For shared-frailty models, the data are organized into *G* groups with the *i*th group consisting of *n*_*i*_ observations, *i* = 1*, …, G*. From Therneau and Grambsch [20], estimation of $\theta$ takes place via maximum profile log likelihood. For fixed$\theta$, estimates of $\beta$ and *ν*_1_*, …, ν*_*G*_ are obtained by maximizing

7 $$\begin{aligned} & \log L\left( \theta \right) = \log L_{Cox} \left( {\beta , v_{1} , \ldots , v_{G} } \right) \\ & \quad + \mathop \sum \limits_{i = 1}^{G} \left[ {\frac{1}{\theta }\left\{ {v_{i} - \exp \left( {v_{i} } \right)} \right\} + } \right.\left( {\frac{1}{\theta } + D_{i} } \right)\left\{ {1 - \log \left( {\frac{1}{\theta } + D_{i} } \right)} \right\} - \frac{\log \theta }{\theta } + \left. {\log {\Gamma }\left( {\frac{1}{\theta } + D_{i} } \right) - \log {\Gamma }\left( {\frac{1}{\theta }} \right)} \right] \\ \end{aligned}$$

where *D*_*i*_ is the number of death events in group *i*, and log*L*_Cox_($\beta$*; ν*_1_*, …, ν*_*G*_) is the standard Cox partial log likelihood, with the *νi* treated as the coefficients of indicator variables identifying the groups. That is, the *j*th observation in the *i*th group has log relative hazard $x\beta$ + *ν*_*i*_. The estimate of the frailty parameter, $\widehat{\theta }$, is chosen as that which maximizes log*L*($\theta$). The final estimates of ${\varvec{\beta}}$ are obtained by maximizing log*L*($\widehat{\theta }$) in ${\varvec{\beta}}$ and the ${\nu }_{i}$.

The estimated variance–covariance matrix of $\widehat{{\varvec{\beta}}}$ is obtained as the appropriate submatrix of the variance matrix of ($\widehat{{\varvec{\beta}}}$*,* ${{\widehat{{\varvec{v}}}}_{1},\boldsymbol{ }\dots ,{\widehat{{\varvec{v}}}}_{{\varvec{G}}}\boldsymbol{ })}_{,}$ and that matrix is obtained as the inverse of the negative Hessian of log*L*($\widehat{{\varvec{\theta}}}$). Therefore, standard errors and inference based on $\widehat{{\varvec{\beta}}}$ should be treated as conditional on$\theta = \widehat{{\varvec{\theta}}}$.

The likelihood-ratio test statistic for testing *H*0: *θ* = 0 is calculated as minus twice the difference between the log likelihood for a Cox model without shared frailty and log*L*($\widehat{{\varvec{\theta}}}$) evaluated at the final ($\widehat{{\varvec{\beta}}}$*,*${{\widehat{{\varvec{v}}}}_{1},\boldsymbol{ }\dots ,{\widehat{{\varvec{v}}}}_{{\varvec{G}}}\boldsymbol{ })}_{,}$.

### Accounting for complex survey design

The DHS surveys are designed using a complex survey design that involves stratification, clustering, and weighting to ensure that the survey sample is representative of the population of interest. Frailty models can account for clustering by including a random effect or frailty term in the model that captures the unobserved heterogeneity between the clusters.

### Ethics approvals

Ethics approval was not required for this study since the data is secondary and is available in the public domain. More details regarding MDHS data and ethical standards are available at: http://goo.gl/ny8T6X

## Results

### Descriptive statistics

A total of 17,286 live births born during the 5 years preceding the survey. Table 1 provides a bivariate comparison of characteristics between those who died and those censored. This comparison only focused on those variables used in the analysis of survival. Maternal age categories were not different between the hazard and censored children except for the age range of 40–49. Among the poor and rich households, there were also variation deaths and censoring unlike the middle class. Both low and high birth weight, Sex of the child, Residence, Birth order, Number of antenatal care visits, Infant breastfed status at birth, had within variation between deaths and censoring.

**Table 1:** Summary results of covariates of time-to-death for under-five children in Malawi, 2015/16 Malawi Demographic and Health Survey

Variable	Category	Hazard (%)	Censored (%)	${\chi }^{2}$ *P*-value
Maternal age	15–19	85 (10.3%)	1,161 (7.0%)	0.0005
	20–29	417 (50.6%)	8,893 (54.0%)	0.0597
	30–39	245 (32.1%)	5,283 (29.7%)	0.168
	40–49	77 (9.3%)	1,125 (6.83%)	0.0070
Maternal age at first birth	≥ 20 years	259 (31.4%)	4,989 (30.3%)	0.964
	< 20 years	565 (68.6%)	11,473 (69.7)	0.9919
Maternal educational status	No education	101 (12.3%)	2060 (12.5%)	0.8704
	Primary school	573 (69.5%)	10,883 (66.1%)	0.04615
	Secondary school	141 (17.1%)	3242 (19.7%)	0.07537
	Tertiary	9 (1.1%)	277 (1.7%)	0.2474
Wealth Index	Poor	405 (49.2%)	7247 (44%)	0.00428
	Middle	150 (18.2%)	3219 (19.6%)	0.3629
	Rich	269 (32.6%)	5996 (36.4%)	0.03044
Sleep in treated net/use of treated mosquito net	Yes	409 (49.6%)	7243 (44%)	0.5176
	No	415 (50.4%)	9219 (56%)	0.5176
Birth weight of the child	Low Birth weight	109 (13.2%)	1660 (10.1%)	0.00441
	High Birth Weight	715 (86.8%)	14,802 (89.9%)	0.00441
Infant breastfed status at birth	Immediately	239 (29%)	10,324 (62.7%)	0.0001
	Not immediately	585 (71%)	6138 (37.3%)	0.0001
Place of birth	Home	74 (9%)	1039 (6.3%)	0.00294
	Public health facility	639 (77.5%)	13,073 (79.4%)	0.2129
	Private health facility	100 (12.1%)	2120 (12.9%)	0.57
Sex of the child	Male	369 (44.8%)	8230 (50%)	0.00391
	Female	455 (55.2%)	8232 (50%)	0.00391
Residence	Urban	714 (86.7%)	13,806 (83.9%)	0.0376
	Rural	110 (13.3%)	2656 (16.1%)	0.0376
Birth order	< 3	410 (49.8%)	7468 (45.4%)	0.0149
	3–4	196 (23.8%)	4990 (30.3%)	0.0001
	> 4	218 (26.5%)	4004 (24.3%)	0.1771
Number of antenatal care visits	< 4	218 (26.5%)	6342 (38.5%)	0.00441
	≥ 4	606 (73.5%)	10,120 (61.5%)	0.0001

### Bivariate analysis of survival in under-five children using Kaplan–Meier survival analysis

**Fig. 2:**
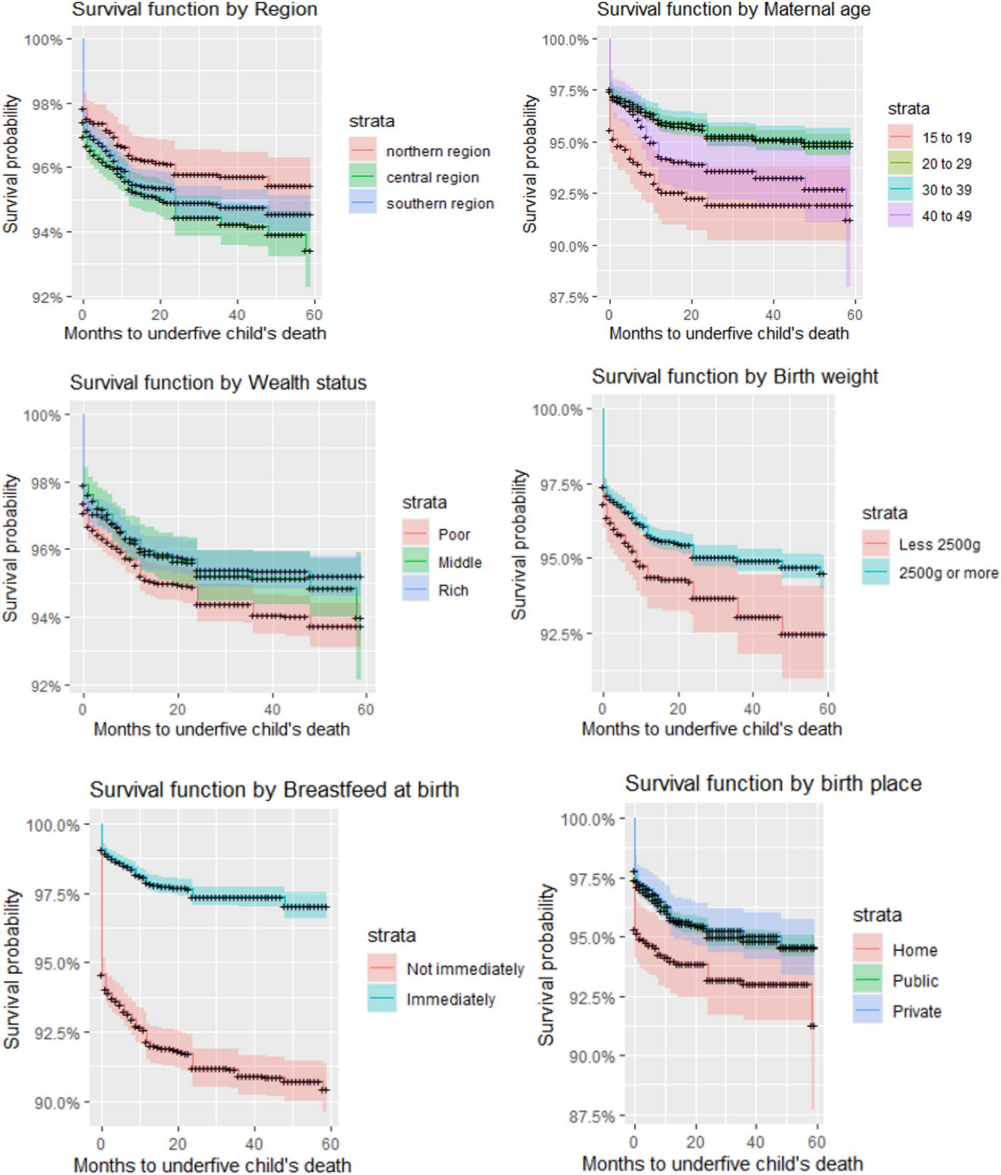

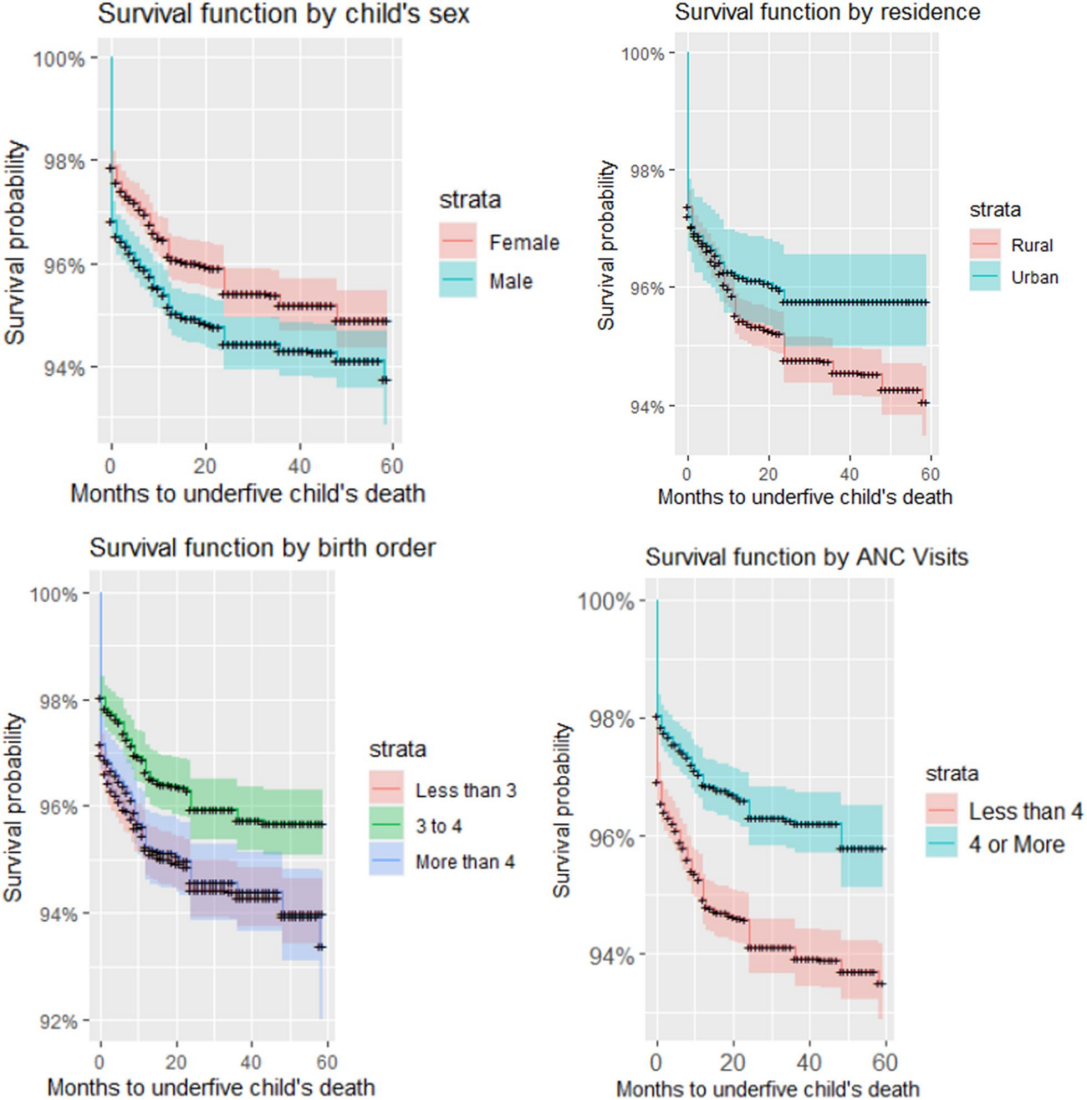
Kaplan–Meir survival estimate of under-five children

Correlates of the under-five child mortality were explored further using a bivariate analysis. The Kaplan–Meier Survival curves are in Fig. 2. Children in the northern region of Malawi were more likely to die than in Southern regions. Central region was the least in child’s likelihood to survive. The maternal age had varied effects on the child survival. The Kaplan–Meier shows that the teenage mothers were at risk of losing the children, similarly, under-five children of late motherhood were at high risk of death. Those mothers within a high fertility block, that is age of 20 to 39 were most likely to have their children survive their under-five period. Socioeconomic status reduced the survival probability of a child. Children from mothers in middle to rich households were more likely to survive. There was apparent difference in survival of children by weight of a child at birth. Birth weight of less that 2500g reduced the survival probability by a large margin when compared with those born with weight of 2500g or more. Timing of breastfeeding at birth was a key factor in child mortality outcomes. There was a huge gap of survival probability between those who immediately breastfed their child and those that did but not immediately. Comparing between various places of birth, the private hospitals contribute highly to child survival, followed by public hospitals and lastly the home delivery. Female children were more likely to survival than male children do, just as the rural and urban, respectively. Birth order was another important factor in child mortality. Looking at birth orders of the ranges less than 3, 3 to 4 and above 4, the optimal birth order for increased survey likelihood was 3 to 4. Low and higher birth orders were associated with low survival probability. A very unusual finding was for the number of visits to antenatal clinic (ANC). Less than 4 clinical visits were associated with high survival probability than more clinics.

### Comparison of various models

The study made several assumptions about the baseline hazard parametric distribution. The Gompertz baseline distribution with gamma frailty distribution had the best-fit model based on the information criterion. Inverse Gaussian frailty distribution with a lognormal baseline hazard distribution did not converge. Given the lowest AIC value, Gompertz's baseline distribution with gamma frailty distribution was the best model (Table 2).

**Table 2:** Model comparison with different distributional assumptions

Baseline distribution	Frailty	AIC	BIC
Exponential	Gamma	859.372	1004.097
Weibull	Gamma	857.1418	1008.440
Loglogistic	Gamma	831.3592	982.6595
Lognormal	Gamma	826.649	977.9571
Gompertz	Gamma	744.2175	895.5191
Exponential	Inverse Gaussian	886.8715	1031.589
Weibull	Inverse Gaussian	860.3382	1011.637
Loglogistic	Inverse Gaussian	830.2691	981.5695
Lognormal	Inverse Gaussian	nc*	nc*
Gompertz	Inverse Gaussian	744.2187	895.5191

^*^Not convergent

### Multivariate analysis of survival in under-five children

To recognize the potential significant factors for under-five children’s mortality a parametric cluster-level shared frailty survival model was fit. The value of the Gompertz distribution shape parameter (gamma) in the baseline hazard distribution was (ρ =  − 0.106, 95%CI: − 0.1231, − 0.0894). This negative value points that the hazard of death among under-five children declined exponentially with aging of under-five children increase. The dependency (heterogeneity) of under-five children in the same cluster estimated by the model was not statistically significant with a value theta (θ = -13.415), and the dependency within-cluster was negligible.

After controlling cluster-level frailty, the results from Gompertz parametric baseline hazard distribution revealed that the age of a woman, antenatal visits, access to mosquito nets, immediate breastfeeding at birth and sex of a child were statistical predictors of under-five child survival. The hazard of death among children born from mothers aged 20 to 29 was 2.1 (HR = 2.1, 95% CI: 1.0181–4.5645). For mothers aged 30 to 39 the risk was 3.8 times (HR = 3.75, 95% CI: 1.5004–9.3853), and the risk was even higher, 11 times (HR = 11.4, 95% CI: 3.8139 − 34.1936) in aged mothers compared to teen mothers (15 to 19). Those who attended antenatal care visits less than 4 times were 3 times at more risk of death when compared with those who met the advocated minimum number of visits (HR = 3, 95% CI: 1.9782–4.5720). The estimated hazard of death among under-five children who were sleeping under mosquito nets lowered by 68% as compared to those who did not use mosquito nets (HR = 0.32, 95%CI: 0.2161–0.4706). In the same way, those who were breastfed immediately after delivery had a 79% lower risk of death compared to those took longer to first breastfeed (HR = 0.21, 95% CI: 0.1432–0.3002). The estimated hazard of death among male under-five children was lowered by 33% as compared to female infants (HR = 0.67, 95%CI: 0.90–0.97). From poverty perspective, the rich household had a 35% reduced risk of infant deaths (HR = 0.65, 95% CI: 0 0.4362, 0.9824) (Table 3).

**Table 3:** Results of multivariable parametric Gompertz distribution cluster-level shared frailty survival regression model among under-five children in Malawi,

Variable	Hazard Ratio	Std Error	*P*-value	95% LCL	95% UCL
Age of mother					
20–29	2.1557*	0.8251	0.0450	1.0181	4.5645
30–39	3.7526*	1.7551	0.0050	1.5004	9.3853
40–49	11.4197*	6.3899	0.0001	3.8139	34.1936
Birth Order					
3–4	0.7407	0.1900	0.2420	0.4481	1.2244
4 or more	0.4917*	0.1863	0.0610	0.2339	1.0334
Household size	0.8558	0.0441	0.003	0.7736	0.9468
Sex of household head	0.7650	0.1851	0.268	0.4761	1.2291
Antenatal visits					
Less than 4 ANC visits	3.0074*	0.6427	0.0001	1.9782	4.5720
Socioeconomic status (Poverty)					
Middle	1.0783	0.2558	0.7510	0.6774	1.7166
Rich	0.6547*	0 .135577	0.0410	0 .4362	0.9824
Use of mosquito nets	0.3189*	0.0633	0.0001	0.2161	0.4706
Early breast feeding at birth	0.2074*	0.0391	0.0001	0.1432	0.3002
Place of Birth					
Public hospital	0.6755	0.2305	0.2500	0.3461	1.3184
Private hospital	0.8918	0.3510	0.7710	0.4123	1.9289
Birth weight					
Hight birthweight	0.6719	0.1686	0.1130	0.4109	1.0986
Place of residence					
Residence	0.5300*	0.1726	0.0410	0.2800	1.0033
Sex of child					
Male	0.6658*	0.1217	0.0260	0.4653	0.9525
Education level of mother					
Primary	0.9088	0.2644	0.7420	0.5138	1.6073
Secondary	1.1236	0.3977	0.7420	0.5615	2.2484
Tertiary	1.03E-12	6.80E-07	1.0000	0.0000	
Occupation					
Mother	1.8247	0.4044	0.007	1.1818	2.8172
Father	0.6392	0.2113	0.176	0.3344	1.2218
Water and sanitation					
Portable water	0.8138	0.2004	0.403	0.5022	1.3187
Improved toilet	1.0969	0.1997	0.611	0.7677	1.5673
Region					
Central	0.6333	0.1634	0.0770	0.3819	1.0500
Southern	0.8655	0.1965	0.5250	0.5547	1.3506
Intercept	0.3907	0.2534	0.1470	0.1096	1.3928
Gamma	− 0.1062*	0.0086	0.0001	− 0.1231	− 0.0894
Frailty theta	− 13.389	435.670	0.9850	− 867.5	840.6
Frailty Kendall’s tau (theta)	0.000*	0.001		–	
Log likelihood	− 349.113				
Chi-square	199.49*		0.0001		

^*^Significant at *P* < 0.05 levels

## Discussion and conclusions

The aim of this research was to identify the factors that affect the mortality rate of under-five children in Malawi. The study utilizes recent Demographic and Health Survey data from 2015/16 and employs a cluster-based Shared frailty analysis to evaluate the survival status and factors affecting under-five mortality in Sub-Saharan Africa. The study had two distinct stages. The first stage involved analyzing the distribution of child deaths based on various factors. The second stage involved using a statistical method called Shared Frailty to examine the probability of survival through multivariate regression. The study also compared the performance of different models and selected the most effective one using the Bayesian Criterion Method. The results of the study revealed that out of 17,286 under-five children, 824 (4.77%) died before reaching their fifth birthday. The major factors influencing under-five child mortality were socio-demographics, such as the age of the mother at delivery, access to mosquito nets, antenatal visits, breastfeeding, and the sex of the child.

There is evidence to suggest that mother’ age is associated with child survival. Studies have shown that children born to younger mothers (under the age of 20) are at a higher risk of dying before the age of five than those born to mothers in their 20s and 30s. This may be due to a lack of physical and emotional maturity, as well as limited access to education, healthcare, and other resources. Additionally, older mothers (over the age of 35) may also have an increased risk of giving birth to children with health complications, which can contribute to higher mortality rates [10, 11]. Our findings show that the hazard ratio increases with age of the mother. Infants from younger mothers were more likely to survive than from older mothers. A number of reasons could explain this. Younger mothers tend to be in better physical and mental health than older mothers, which can increase the chances of a healthy pregnancy and delivery. Younger mothers may have more access to prenatal care and education, which can improve the health of both the mother and the baby. Younger mothers are also more likely to have more energy and resilience to cope with the physical and emotional demands of parenting, which can lead to better outcomes for the baby. There are more likely to have more support from family and friends, which can provide emotional and practical help during the pregnancy and after the baby is born. In addition, young mothers are less likely to have chronic health conditions or other health issues that could increase the risk of complications during pregnancy and delivery.

The study finds a positive association of mosquito net use and infants’ deaths. The use of mosquito nets has shown to be an effective intervention in reducing infant mortality. Mosquito nets can protect infants and their families from malaria, which is a major cause of death among children under the age of five in many developing countries. Mosquito nets provide a physical barrier between the person sleeping under the net and the mosquitoes, reducing the chances of being bitten and contracting malaria. Insecticide-treated mosquito nets (ITNs) also have an insecticide that kills or repels mosquitoes, which further reduces the risk of infection. The use of mosquito nets can also reduce the rate of anemia in children and pregnant women, which is a common complication of malaria. Malaria-related deaths account for a significant proportion of infant mortality in sub-Saharan Africa, and the use of mosquito nets is an asset to reduce infant mortality rates [1].

The hazard ratio of 0.2073 for early initiation of breastfeeding suggests that there is a protective effect of breastfeeding on child deaths. Specifically, the hazard ratio represents the relative risk of the outcome (child deaths) for the exposed group (those who initiated breastfeeding early) compared to the unexposed group (those who did not initiate breastfeeding early). In this case, the hazard ratio of 0.2073 suggests that the risk of child deaths is approximately 80% lower in infants who were breastfed within the first hour after birth than those who were not. This finding is in line with existing research on the benefits of early initiation of breastfeeding for child survival [24]). Breastfeeding provides essential nutrients and antibodies to infants, which can help to protect them from infection and disease. Additionally, early initiation of breastfeeding links to reduced risk of neonatal infection and improved cognitive development in infants. Overall, this result highlights the importance of promoting and supporting early initiation of breastfeeding as a strategy for reducing child deaths.

Our result in line with other studies. For example Lartey et al. [9] show a significant effect of household wealth on under-five survival. The hazard ratio of 0.65 for mothers from rich households suggests that there is a reduced risk of child deaths among this group compared to mothers from less affluent households. The risk of child deaths is approximately 35% lower among mothers from rich households compared to those from less affluent households. This finding could be due to several factors, such as access to better healthcare and nutrition, as well as more resources for maternal and child care. It is also possible that mothers from rich households may have more knowledge and education about child care and health. Nevertheless, this result highlights the importance of addressing socioeconomic disparities in child health and mortality. There is need to strategically target the poor, such us ensuring proper stocking of essential resources ranging from equipment and human capital in health facilities that serve the poor and ensuring that quality maternal services are accessible for the poor population.

The hazard ratio of 0.53 for urban residents suggests that there is a reduced risk of child deaths among this group compared to rural residents. Thus, the risk of child deaths is approximately 47% lower among urban residents compared to rural residents. This finding could be due to several factors, such as access to better healthcare and nutrition, as well as more resources for maternal and child care. Urban areas often have better infrastructure and access to services, such as hospitals, clinics, and nutrition programs, which can improve health outcomes for children. The high demand for professional health workers in urban areas tend to pull them towards urban [4]. Hence, it would require a set of good incentives to keep professional health workers such as doctors in rural health facilities. Additionally, urban residents may have more knowledge and education about childcare and health, which can also contribute to better health outcomes. This result highlights the importance of addressing disparities in child health and mortality between urban and rural areas. Policies and programs that aim to improve maternal and child health in rural areas may help to reduce child deaths and improve health outcomes for all children.

## Key conclusions and limitations

Based on the findings of this study, it is evident that infant mortality rates decline as under-five children age, highlighting a significant challenge in ensuring the survival of newborns. Factors such as maternal age, birth order, socioeconomic status, utilization of mosquito nets, early initiation of breastfeeding, geographic location, and the sex of the child play crucial roles in predicting under-five mortality rates. In light of these compelling findings, it is imperative for public health strategies to prioritize interventions targeting these identified predictors to, further, mitigate under-five mortality rates. Recommendations entail the implementation of comprehensive maternal and child health initiatives aimed at imparting crucial knowledge to mothers regarding the significance of early breastfeeding initiation and the adoption of mosquito net usage, particularly in regions vulnerable to vector-borne diseases. Moreover, accessible healthcare services must extend to marginalized communities to address socio-economic disparities that perpetuate differential access to essential resources and healthcare, thereby ensuring equitable opportunities for all children to thrive beyond infancy and early childhood.

The key limitation of the study is that it was not possible to separate deaths induced by medical personnel. Sometimes the medical experts allow for the death of a child in order to save the life of the mother. This data is not captured by DHS studies. Furthermore, in reinforce the external validity of our findings, further research can be conducting using a pooling of DHS cross-sections from various countries and across the years.

## Data Availability

The data used for this study is publicly available at https://dhsprogram.com/methodology/survey/survey-display-483.cfm
